# ORANGE family proteins: multifunctional chaperones shaping plant carotenoid level, plastid development, stress tolerance, and more

**DOI:** 10.1186/s43897-025-00169-9

**Published:** 2025-05-09

**Authors:** Emalee Wrightstone, Lilin Xu, Sombir Rao, Abhijit Hazra, Li Li

**Affiliations:** 1 Plant Breeding and Genetics Section, School of Integrative Plant Science, Cornell University, Ithaca, NY 14853 USA; 2 Robert W. Holley Center for Agriculture and Health, USDA-Agricultural Research Service, Cornell University, Ithaca, NY 14853 USA; 3 Horticulture Section, School of Integrative Plant Science, Cornell University, Ithaca, NY, 14853, USA

**Keywords:** DnaJE1, Chaperones, OR proteins, Carotenoid accumulation, Chloroplast; chromoplast, Stress resilience

## Abstract

ORANGE (OR) family proteins are DnaJE1 molecular chaperones ubiquitous and highly conserved in all plant species, indicating their important roles in plant growth and development. OR proteins have been found to exert multiple functions in regulating carotenoid and chlorophyll biosynthesis, plastid development, and stress tolerance, with additional functions expected to be discovered. As molecular chaperones, OR proteins directly influence the stability of their target proteins via their holdase activity and may perform other molecular roles through unknown mechanisms. Exploration of OR has uncovered novel mechanisms underlying core plant metabolism pathways and expanded our understanding of processes linked to plastid development. Continued investigation of OR family proteins will not only reveal new functions of molecular chaperones but also provide pioneering tools for crop improvement. Thus, OR family proteins offer a distinctive opportunity to comprehend molecular chaperones in modulating various metabolic and developmental processes and exemplify the importance of chaperones in crop development and adaptability. This review briefly details the history of OR family proteins, highlights recent advancements in understanding their myriad of functions, and discusses the prospects of this fascinating group of chaperones towards generating innovative, more nutritious, and resilient crops alongside other agronomically important traits.

## Introduction

**Fig. 1:**
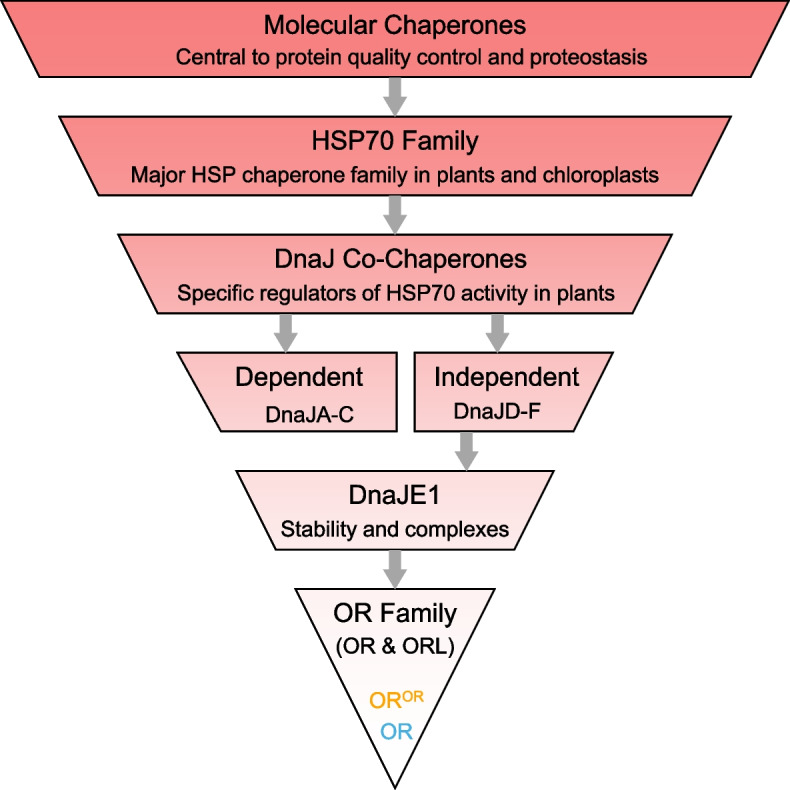
Schematic relationship between molecular chaperones and the ORANGE protein family. Molecular chaperones cover a diverse group of protein families with the common role of regulating protein quality control (PQC) and proteostasis. The HSP70 (Heat shock protein 70) family plays large roles in PQC in plants, particularly in chloroplasts. DnaJ co-chaperones provide HSP70 target specificity via interacting with both HSP70 and their targets. Classes DnaJA-C depend on HSP70 for functions. The closely related classes DnaJD-F evolved from the former and appear to regulate PQC independent of HSP70 proteins. The DnaJE1 class is of particular interest for the involvement of its members in chloroplast protein stability and complex formation. One such member is the ORANGE (OR) family, consisting of OR in algae and higher plants with ORANGE-like (ORL) appearing only in higher plants. Two isoforms of OR, termed OR (wild type) and OR^OR^ (mutant including OR^His^) are of noteworthy study because of their specific roles in regulating carotenoid levels and chromoplast biogenesis

Molecular chaperones are a group of diverse proteins found in prokaryote and eukaryote lineages (Hartl et al. 2011; Rebeaud et al., 2021). They are present in the cytoplasm, mitochondria, endoplasmic reticulum, plastids, and nuclei, with central roles in protein quality control (PQC) and proteostasis through refolding denatured proteins, assisting protein degradation, promoting protein transport, and aiding protein maturation (Hartl et al. 2011; Saibil 2013) (Fig. 1). Many unrelated chaperone families are termed “heat shock proteins” (HSPs) due to their discovery under heat stress conditions (Hartl et al. 2011), although they are not always heat-induced (Park & Seo 2015). HSPs and other chaperone protein families play key roles in maintaining cellular PQC through their interactions with target proteins (Berka et al. 2022; Rosenzweig et al. 2019).

In plants, the major HSP families are small HSPs, HSP40, HSP60, HSP70, HSP90, and HSP100 (Wang et al. 2004). HSP70 chaperones play large roles in PQC and notably influence many chloroplast pathways (Pulido et al. 2017; Schroda et al. 1999; Shi & Theg 2010) (Fig. 1). The specificity of HSP70 protein targets is provided by their DnaJ co-chaperones (Pulido & Leister 2018; Pulido et al. 2013; Rosenzweig et al. 2019) (Fig. 1). Named after the first characterized member from *Escherichia coli* (Liberek et al. 1991), a diverse range of DnaJ proteins have subsequently been identified in plants (Luo et al. 2023; Wang et al. 2015a; Zagari et al. 2017; Zhang et al. 2021).

DnaJ co-chaperones are classified into six major groups as HSP70 dependent or independent (Pulido & Leister 2018) (Fig. 1). The three canonical groups of DnaJ proteins (DnaJA-C) all contain a conserved ~ 70 amino acid sequence termed the J-domain and are essential in the HSP70-dependent process by recognizing and delivering unfolded protein targets to HSP70 (Barriot et al. 2020; Kampinga & Craig 2010). This hierarchy allows a small number of HSP70 proteins to regulate PQC through a relatively larger number of diverse DnaJ co-chaperone proteins, each of which in turn interact with different protein targets. Interestingly, some DnaJ co-chaperones have either less conserved (DnaJD) or entirely absent (DnaJE1 - 2/F) J-domains and are thought to act independently of HSP70. DnaJD is a highly diverse class with some members indirectly influencing HSP70 pathways. DnaJF has a few uncharacterized members containing a DnaJA/B-like C-terminal domain for protein binding and appears to be co-expressed with nuclear- and cytosolic-targeted proteins (Pulido & Leister 2018). DnaJE chaperones retain the DnaJA-type zinc finger domain of four CxxCxGxG repeats enabling protein–protein interactions and either lack (DnaJE1) or have (DnaJE2) a glutaredoxin-like (GRL) domain. DnaJE1 proteins are primarily expressed in green tissues and their core roles are associated with preventing protein aggregations and impacting protein complex stability (Kampinga & Craig 2010; Pulido & Leister 2018) (Fig. 1).

One interesting DnaJE1 family is the ORANGE (OR) family that contains OR and OR-LIKE (ORL) (Fig. 1). OR was first identified from a cauliflower (*Brassica oleracea* var. *botrytis*) mutant with orange-colored curd that accumulates high levels of β-carotene (Li et al. 2006; Li et al., 2001; Lu et al. 2006). The mutant *BoOr*^*MUT*^ contains a *copia*-like LTR retrotransposon in exon 3, causing alternatively spliced transcripts which promote carotenoid accumulation and chromoplast biogenesis (Lu et al. 2006). Later discovery of a ‘golden’ single nucleotide polymorphism (SNP) of *CmOr* in orange flesh melon (*Cucumis melo*) further signifies the important role of *Or* orange alleles (*Or*^*ORANGE*^ or *Or*^*OR*^*)* in carotenoid accumulation (Chayut et al. 2017; Tzuri et al. 2015).

Following delineation of *Or* in orange cauliflower and melon fruit, functions of the family proteins have been extensively investigated in regulating carotenoid accumulation (Chayut et al. 2017; Jaramillo et al. 2022; Yuan et al. 2015a; Zhou et al. 2015), plastid development (Chayut et al. 2017; Hitchcock et al. 2023; Sun et al. 2023, 2020b, 2019) and abiotic stress tolerance (Ali et al., 2022; Jung et al., 2021; Kang et al. 2023; Kim et al., 2021). Moreover, their potential in crop biofortification has been well documented in the literature (Ahrazem et al. 2022; Bai et al. 2016; Berman et al. 2017; He et al. 2022; Jaramillo et al. 2022; Lopez et al. 2008; Sun et al., 2021; Yazdani et al. 2019). OR chaperones represent a highly studied DnaJE1 family with great potential in improving agronomic traits (Feder et al. 2019; Hitchcock et al. 2023; Kang et al. 2017b; Kim et al. 2018; Liang & Li 2023; Liang et al. 2023; Osorio 2019; Watkins & Pogson 2020; Zhou et al. 2008).

This review summarizes the history of these unique OR family proteins, highlights the most recent advances in understanding their multifunctional roles, and discusses their potential new functions in modulating plant growth and development. The contributions of DnaJE1 chaperone proteins towards crop biofortification and agronomically important traits are also postulated.

### Structure of OR proteins

OR family proteins are highly conserved in higher plants and algae (Lu et al. 2006; Tzuri et al. 2015). As DnaJE1 members, OR proteins lack the J-domain and retain the DnaJA-type zinc finger domain. They primarily localize to plastids (Kang et al. 2017a; Lu et al. 2006; Zhou et al. 2015) but also are found in the nuclei of etiolated seedlings (Chen et al., 2021; Sun et al. 2019; Zhou et al. 2011) due to a nuclear localization signal following the chloroplast transit peptide (Sun et al. 2016). Two predicted transmembrane domains preceding the zinc finger domain suggest a membrane localization and that proper orientation of the N- and C-terminals may be required for OR-mediated chaperone activity (Welsch et al. 2019). Through yeast-two-hybrid interactions the N- and C-terminals of OR were demonstrated to be required for phytoene synthase (PSY) interaction and OR dimerization, respectively (Zhou et al. 2015). PSY is the key rate-limiting enzyme of carotenoid biosynthesis (Zhou et al. 2022) and its activity is positively correlated with carotenoid content (Paine et al. 2005; Welsch et al. 2018). Although the N-terminus of OR does not have a recognized domain, its high conservation suggests that a yet to be identified domain is responsible for interaction with PSY and potentially other protein targets. The DnaJA-type zinc finger domain resides in the C-terminus (Lu et al. 2006) and is the main facilitator of protein–protein interactions. Dimerization of OR is proposed to safeguard itself and its target proteins from proteolysis or perhaps is needed for its chaperone activity (Zhou et al. 2015). Mutations that prevent the proper orientation of the OR N- or C-terminals in cauliflower (Welsch et al. 2019) and melon (Chayut et al. 2017) significantly reduce carotenoid accumulation.

The conserved nature of OR family proteins in land plants brings to question their roles in plant growth and development, functions towards plant fitness and survival, and contributions in plant adaptation to new and changing environments. Interestingly, while OR is highly conserved, the presence of certain alleles is associated with substantial differences in promoting carotenoid accumulation. The *BoOr*^*MUT*^ allele with a retrotransposon in exon 3 causes orange curd cauliflower (Lu et al. 2006). Similarly, orange-leafed *Brassica rapa* contains a small exon 3 insertion leading to a parallel result (L Zhang et al. 2022c ). Other carotenoid accumulating *Or* or *OrL* alleles are a SNP missense mutation that alters an amino acid residue in the translated protein as in the cases of melon (Tzuri et al. 2015) and carrot (Ellison et al. 2018). Existence of these carotenoid accumulating *Or*^*OR*^ alleles provides a distinguishing function of these DnaJE1 chaperones.

### OR in regulating carotenoid accumulation

**Fig. 2:**
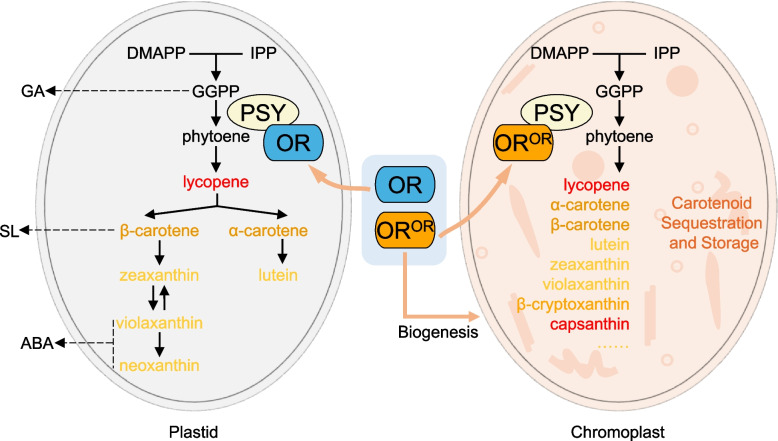
Functions of OR family proteins in carotenoid biosynthesis and carotenoid accumulation in plastids. Both ORANGE family proteins (representing by OR here) and ORANGE^ORANGE^ (OR^OR^) isoforms are chaperones for phytoene synthase (PSY) in plastids and directly regulate carotenoid biosynthesis via modulating the stability of PSY, the key rate limiting and first committed enzyme of carotenoid pathway. OR^OR^ gains an additional function to promote biogenesis of chromoplasts, the specific plastid with strong sink strength for carotenoid sequestration and storage, leading to higher capacity of carotenoid accumulation. Thus, OR^OR^ has dual functions in regulating both carotenoid biosynthesis and chromoplast biogenesis and represents an effective genetic tool for carotenoid biofortification. A simplified carotenoid biosynthesis pathway is outlined. DMAPP, dimethylallyl diphosphate; IPP, isopentenyl diphosphate; GGPP, geranylgeranyl diphosphate. The carotenoid pathway is associated the production of plant hormones of gibberellin (GA) from GGPP, strigolactone (SL) from β-carotene, and abscisic acid (ABA) from violaxanthin and neoxanthin. Some major carotenoids accumulated in chromoplasts are listed

The carotenoid biosynthetic pathway occurs in various plastids such as chloroplasts and chromoplasts (Sun et al. 2018) (Fig. 2). Dimethylallyl diphosphate (DMAPP) and isopentyl diphosphate (IPP) from the plastidial methylerythritol phosphate pathway are used to synthesize geranylgeranyl diphosphate (GGPP), the precursor for carotenoids alongside chlorophylls, plastoquinones, tocopherols, and gibberellins (GA). PSY catalyzes the first committed, rate-limiting step of carotenoid biosynthesis through condensation of two GGPPs into 15-*cis*-phytoene. Subsequent reactions form all-*trans*-lycopene that is cyclized to α- or β-carotene and then oxygenated to xanthophylls (Canniffe & Hitchcock 2021; Nisar et al. 2015; Sun et al. 2020a). Cleavage of carotenoids through enzymatic and/or non-enzymatic reactions generates apocarotenoids responsible for the formation of plant signaling metabolites along with the phytohormones abscisic acid (ABA) and strigolactone (SL) (Sun et al. 2020a) (Fig. 2).

The carotenoid profile of green leaf tissues is highly regulated for optimal photosynthetic activity (Domonkos et al. 2013; Sandmann 2021; Sun et al. 2022). However, non-green organs including fruits, flowers, and tubers display tremendously diverse carotenoid levels and compositions, both between and within species (Hermanns et al. 2020; Yuan et al. 2015b). Decades of extensive research have uncovered a certain degree of transcriptional, post-translational, epigenetic, and environmental mechanisms governing carotenoid levels and profiles (Bou-Torrent et al. 2015; Liu et al. 2015; Lu & Li 2008; Nisar et al. 2015; Rodriguez-Concepcion et al. 2018; Sun et al. 2022).

OR family proteins primarily regulate carotenoid biosynthesis through post-translationally stabilizing PSY and potentially other pathway enzymes via protein–protein interactions (Chayut et al. 2017; Park et al. 2016; Welsch et al. 2018; Zhou et al. 2015) (Fig. 2). Alterations in PSY stability or activity are well recorded to influence carotenoid content (Cao et al. 2019; Maass et al. 2009; Paine et al. 2005). The importance of PSY and OR interactions in carotenogenesis extends beyond higher plants. While the algae *Dunaliella salina* CCAP 19/18 accumulates β-carotene from this interplay, *Dunaliella* sp. FACHB- 847 does not due to reduced interaction with OR despite high PSY activity (Liang et al. 2023). Further, association between PSY and OR may also be important for the creation of yet unknown apocarotenoid signals, which when reduced limits plastid and chlorophyll formation (Hou et al. 2024). PSY protein level is balanced between stability from OR chaperones and proteolysis through the Clp-mediated pathway (D'Andrea et al. 2018; Welsch et al. 2018).

In addition to regulating PSY, OR^OR^ from orange alleles gains novel functions that significantly stimulate carotenoid accumulation by inducing chromoplast biogenesis (Lu et al. 2006; Tzuri et al. 2015) (Fig. 2). Following identification of *BoOr*, homologs were sought in other orange-colored fruit tissues (Cuevas et al., 2009; Tzuri et al. 2015). Melon has two *CmOr* alleles differentiated by a SNP which translates to either Arg^108^ or His^108^ and is responsible for non-orange and orange flesh fruit, respectively. Plants harboring this “golden SNP” allele *ORANGE*^*His*^ (*CmOr*^*His*^) contains comparable *PSY* gene expression, PSY protein levels, and carotenogenic metabolic flux to those with the wild-type allele (*CmOr*^*Arg*^) (Tzuri et al. 2015). Akin to *BoOr*^*MUT*^, *CmOr*^*His*^ has a new function to induce chromoplast biogenesis and support accumulation of carotenoids (Chayut et al. 2017).

To examine whether the “golden SNP” is broadly significant, a genetic mimic was created by mutating Arabidopsis *AtOr* to *AtOr*^*His*^ (Yuan et al. 2015a). Overexpression of *AtOr*^*His*^ induces chromoplast formation with increased β-carotene accumulation in Arabidopsis callus and tomato fruit tissues (Sun et al. 2020b; Sun et al., 2021; Yazdani et al. 2019; Yuan et al. 2015a). A similarly acting missense mutation in *OrL* of orange carrots (*Daucus carota* L. var. *sativa*) termed *DcOr3*^*Leu*^ amasses large amounts of carotenoids relative to low carotene carrots with the *DcOr3*^*Ser*^ allele. *DcOr3*^*Leu*^ knockout in orange carrot roots reduces chromoplast formation and generates yellow-colored roots (Coe et al. 2021; Ellison et al. 2018).

There is clear evidence that the OR family proteins play important roles in post-translationally modulating PSY to regulate carotenoid biosynthesis in plants (Chayut et al. 2017; Liang & Li 2023; Zhou et al. 2015). The OR^OR^ gain of function isoforms possess dual functions in not only regulating PSY stability for carotenoid biosynthesis but also promoting chromoplast formation with enhanced sequestration and storage strength to greatly increase carotenoid accumulation (Chayut et al. 2017; Sun et al. 2020b; Sun et al., 2021; Tzuri et al. 2015; Yazdani et al. 2019) (Fig. 2).

### OR and plastid development

**Fig. 3:**
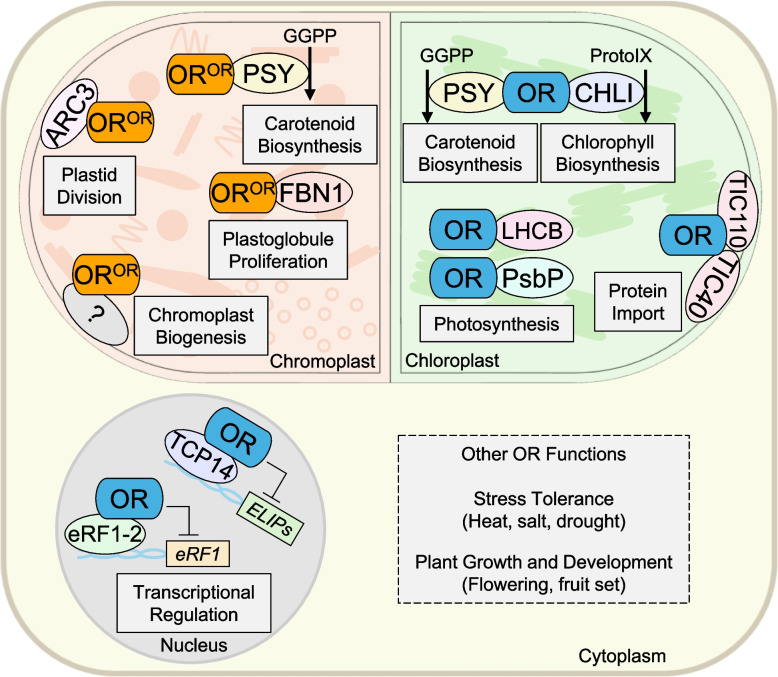
Overview of OR functions. Targets and associated processes of ORANGE family proteins (representing by OR here) and ORANGE^ORANGE^ (OR^OR^) isoforms are noted in the chromoplast, chloroplast, and nucleus, which are experimentally verified. Other OR functions with undefined mechanisms are listed. ARC3, accumulation and replication of chloroplasts 3; CHLI, magnesium chelatase subunit I; eRF1-2, eukaryotic release factor 1–2; FBN1, fibrillin 1; LHCB, light-harvesting chlorophyll *a/b* binding; PsbP, oxygen-evolving enhancer protein; PSY, phytoene synthase; TCP14, teosinte branched1/cycloidea/proliferating cell factor 14; TIC40/TIC110, translocon at the inner chloroplast envelope 40/110. *ELIP*s, *Early Light-Inducible Proteins*; *eRF1*, *Eukaryotic Release Factor1*

Plant carotenogenesis occurs in plastids, in order of increasing production capabilities from etioplasts, amyloplasts, chloroplasts, to chromoplasts (Sun et al. 2018). Chloroplasts primarily accumulate carotenoids in thylakoid membranes (Sun et al. 2020a) and tightly control the proportions alongside chlorophylls for optimal photosynthesis (Sun & Li 2020). Chromoplasts are developed from various plastids (Sun et al. 2018) and are the major plastid imparting color to the flowers, tubers, and fruits (Egea et al. 2010; Hermanns et al. 2020; Yuan et al. 2015b). While many elements participate in chromoplast development, OR^OR^ represents the only known protein that induces *bona fide* chromoplast biogenesis through yet to be discovered mechanisms (Fig. 3).

Loss of OR^OR^ function limits chromoplast formation and subsequent carotenoid accumulation. For example, the melon *low-β* mutant has a truncated CmOR^His^ protein and produces β-carotene deficient fruit without chromoplasts (Chayut et al. 2017, 2021; Zhou et al. 2023). Additionally, *DcOr3*^*Leu*^ knockout in orange carrots reduces root chromoplast biogenesis and restricts carotenogenesis to the point of yellow roots (Zhang et al., 2022b). Interestingly, in contrast to wild type OR that shows no effect on chromoplast biogenesis, OR^His^ mimics stimulate chromoplast biogenesis with greatly enhanced β-carotene accumulation in Arabidopsis callus and tomato fruit (Sun et al. 2020b; Yazdani et al. 2019; Yuan et al. 2015a).

Orange curd cauliflower and orange flesh melon are noted to have an abnormal one or two large chromoplasts per cell (Chayut et al. 2017; Paolillo et al., 2004), suggesting a relationship between OR^OR^ proteins and plastid division. *AtOr*^*His*^ callus shows the same phenomenon confirming a conservation of this restriction in other species (Yuan et al. 2015a). Plastid division needs physical interaction between ARC3 and PARC6 factors (Zhang et al. 2016). Recent work indicates AtOR^His^, but not AtOR, specifically interacts with ARC3 (Fig. 3) and prevents the association with PARC6 (Sun et al. 2022). Such a restriction on chromoplast number can be relaxed by increased expression of plastid division factors like PDV1 (Sun et al. 2022).

Storage capacity of carotenoids in chromoplasts is directly related to the proliferation of their sequestration substructures (Sun et al. 2018). These are classified into crystalline, fibrillar, globular, membranous, and tubular types (Li & Yuan 2013); each of which has different capacities and may be biased towards specific carotenoid species (Sun et al. 2018). Some studies have explored the relationship between carotenoid biosynthesis, accumulation, and substructure composition. In citrus fruits changes made to plastoglobules, a substructure in globular chromoplasts, create more nonpolar environments favorable to carotenoid storage (Liu et al. 2024). Carrot roots of different colors have different substructures associating carotenoid composition and storage environment (Zhang et al., 2022b).

OR^OR^ proteins in orange curd cauliflower chromoplasts induce condensed whirly multi-layered membrane substructures (Paolillo et al., 2004) and in orange melon fruit form abundant plastoglobules (Jeffery et al., 2012). Fibrillin (FBN) proteins are known to be involved in carotenoid sequestration and storage (Iglesias-Sanchez et al. 2024; Kilcrease et al. 2015). Interaction studies show that FBN1 associates with OR^His^ in melon to stimulate plastoglobule proliferation (Zhou et al. 2023) (Fig. 3) and high OR activity may even alter the substructure landscape (D'Andrea & Rodriguez-Concepcion 2019). Lack of this connection reduces the capacity of membranes for carotenoid production and storage. For example, algae overexpressing *Or*^*His*^ grow larger cells with more plastoglobules and fragmented starch granules relative to *Or* overexpression (Liang et al. 2023; Yazdani et al. 2021). This implies a conserved function of OR^His^ in altering substructure formation.

Beyond the unique functions of OR^His^ related to chromoplast biogenesis, division, and substructure, OR family proteins have been documented to affect various processes for chloroplast biogenesis and development (Chen et al., 2021; Sun et al. 2023, 2019; Yuan et al. 2021a) (Fig. 3). OR proteins coordinately regulate photosynthetic pigment synthesis, affect LHCII assembly, and thylakoid membrane development in chloroplasts (Sun et al. 2023). In green tissues, the ratio of chlorophylls to carotenoids in highly conserved to balance light absorption and photoprotection (Sun & Li 2020). OR chaperones were found to interact with not only PSY, but also CHLI, a component of the magnesium chelatase complex which conducts the first committed step of chlorophyll biosynthesis (Khangura et al. 2020). Loss of OR family function inhibits both chlorophyll and carotenoid production, leading to impaired thylakoid grana stacking (Sun et al. 2023). The connection to photosynthesis is also tied with interactions between OR and chlorophyll *a/b* binding LHCB proteins (Chayut et al. 2021) and PsbP (Kang et al. 2023). It is further supported by higher photosynthetic activity in potatoes overexpressing *Or* (Kang et al. 2023) and reduced expression of photosynthetic genes in the melon *low-β* mutant (Chayut et al. 2021). An important aspect of plastid biogenesis is the import of nascent nuclear-encoded preproteins. Translocation of these polypeptides across the plastid double membrane is through the TIC/TOC translocons (Richardson & Schnell 2020). OR interacts with TIC40 and TIC110 to aid the release of incoming proteins into the plastid stroma (Yuan et al. 2021a).

One surprising feature of OR chaperones is their nuclear localization during photomorphogenesis in de-etiolating seedlings (Chen et al., 2021; Sun et al. 2019). In the nucleus of germinating plants OR interacts with the transcription factor TCP14 and prevents expression of the early light-inducible proteins (ELIPs) (Fig. 3) required for chloroplast biogenesis and chlorophyll stability (Liu et al., 2020; Sun et al. 2019). Mutation of OR’s Lys^58^ residue prevents nuclear localization and subsequent regulation of ELIPs (Chen et al., 2021). Additionally, photomorphogenesis, chloroplast maturation, and chlorophyll biosynthesis are postulated to be controlled by an unknown apocarotenoid signal following a study of mutants with reduced PSY and OR complex formation (Hou et al. 2024). In the nucleus OR may have other targets to influence transcriptional regulation as in the case of BoOR and eRF1-2 interaction to alter *eRF1* genes and petiole length (Zhou et al. 2011), hinting at further OR functions in plant growth (Fig. 3).

### The Or^His^ allele as an effective genetic tool for biofortification

The relationship between carotenoid intake and human health is well documented (Eggersdorfer & Wyss 2018; Rao & Rao 2007). Provitamin A carotenoids such as α- and β-carotene and β-cryptoxanthin produced by plants can be metabolized further in humans to produce vitamin A. Vitamin A deficiency (VAD) primarily impacts young children and pregnant women in developing countries with poor dietary consumption of provitamin A carotenoids, which can cause blindness, increased risks of some cancers, and impaired immune systems (Rodriguez-Concepcion et al. 2018). Biofortification of crops with carotenoids, particularly those with provitamin A compounds, has generated “golden” varieties in maize (Naqvi et al. 2009; Yan et al. 2010), sweet potato (Low & Thiele 2020), banana (Paul et al. 2017), cassava (Sayre et al. 2011), potato (Diretto et al. 2007; Ducreux et al. 2005), rice (Paine et al. 2005; Ye et al. 2000), sorghum (Che et al. 2016), tomato (Romer et al. 2000; Rosati et al. 2000), and wheat (Zeng et al. 2015). OR plays important roles in plant carotenoid biosynthesis and storage structure formation. Since their discovery *Or*^*OR*^ alleles have been thoroughly discussed as promising candidates for the biofortification of staple crops (Li & Van Eck 2007; Sun et al., 2021).

In nature over the course of plant evolution and crop domestication, carotenoid enrichment in crops using *Or* became an accidental happenstance. Some of these resulted from the historic artificial selection of carotenoid accumulating *Or*^*OR*^ alleles as in the cases of orange carrot roots (Coe et al. 2023; Ellison et al. 2018; Shibaya et al. 2022), orange flesh melon fruits (Tzuri et al. 2015), orange leaf *Brassica rapa* (Zhang et al. 2022c), and orange sweet potato tubers (Gemenet et al. 2020). More recently, spontaneous mutations have formed *Or*^*OR*^ alleles as with orange curd cauliflower (Lu et al. 2006) or have been identified in germplasm screening as with the increased expression of *Or* in orange flesh watermelon (Yuan et al. 2021b), orange fruited kiwis (Bhargava et al. 2023), and pink lemons (Lana et al., 2020). However, not all orange-colored fruits or tubers are caused by the OR family proteins. A visually indistinguishable orange flesh watermelon contains mutations in *CRTISO* and accumulates lycopene precursors rather than β-carotene (Fang et al., 2023). Similarly, the orange head *Brassica rapa* Chinese cabbage was mapped to *CRTISO* (Zhang et al., 2013). Additionally, a recessive allele of *ZEP* in orange flesh potato tubers causes a parallel phenotypic outcome (Sulli et al. 2017). Interestingly, the creation and overexpression of *Or*^*His*^ alleles in tomato (Yazdani et al. 2019), potato (Kang et al. 2023; Lopez et al. 2008), sweet potato (Kim et al. 2019; Kim et al., 2021), rice (Jung et al., 2021), and algae (Liang et al. 2023; Yazdani et al. 2021) were all noted to provide a general increase in carotenoid accumulation and support the potential for *Or*^*OR*^ in diverse crop biofortification programs.

Beyond provitamin A carotenoids, some studies have used *Or* to create or improve metabolic flux towards specific carotenoid metabolites in crops. For example, *Camelina sativa* engineered using bacterial phytoene synthase (*crtB*), red algae β-carotene hydroxylase (*CHYB*), green algae β-carotene ketolase (*BKT*), and cauliflower *Or* accumulated astaxanthin, a red colored ketocarotenoid in a seed-specific manner (He et al. 2022). Further, an unspecified mutant of *AtOr* was used to create an apocarotenoid derivative called crocin in *Nicotiana tabacum* when overexpressed along with *Crocus sativus CCD2L* and bacterial β-carotene hydroxylase (*crtZ*) (Ahrazem et al. 2022). Other recent studies have aimed to produce more realistic biofortification attempts such as combining the highly active cassava *OR* with maize *PSY1* (Jaramillo et al. 2022) or expressing *AtOr* in rice (Bai et al. 2016) and maize (Berman et al. 2017) endosperm, reminiscent of Golden Rice 2 (Paine et al. 2005).

Many past efforts to increase β-carotene accumulation in starchy crops have been met with its subsequent degradation and loss during storage (Bollinedi et al. 2019; Che et al. 2016; Ortiz et al. 2018; Schaub et al. 2017), limiting biofortification efficacy. Previously, *BoOr*^*MUT*^ expressed in potato tubers was found to increase β-carotene accumulation before and throughout cold storage as well as enhance its stability compared to wild type controls (Li et al. 2012; Lopez et al. 2008; Van Eck et al. 2010). A recent wholistic approach towards maximizing stable β-carotene accumulation in Arabidopsis seeds was reported. Maize (*Zea mays*) *ZmPSY1* with or without *AtOr*^*His*^ was overexpressed in an Arabidopsis β-carotene hydroxylase (*Atbch2*) knockout. While *ZmPSY1* alone increased seed β-carotene 11-fold, the synergistic effect with *AtOr*^*His*^ improved levels to 65-fold with high storage stability (Sun et al., 2021). This study provides a strong framework and proof of concept for crop biofortification efforts with greatly enhanced carotenoid accumulation and storage stability by simultaneous overexpression of *PSY* and *Or*^*OR*^.

### OR chaperones in plant stress tolerance

Plants are stationary organisms which evolved plasticity to react to and protect themselves from abiotic stress such as nutrients, light, temperature, drought, and salinity (Zhang et al. 2020). In a rapidly changing climate, the additions of genetic regulators that enhance abiotic tolerance are highly favorable (Rivero et al. 2022). Various crop studies overexpressing either native *Or* or mutation *Or*^*His*^ alleles exhibit not only higher carotenoid accumulation, but increased tolerance to heat (Jung et al., 2021; Kim et al. 2019; Kim et al., 2021; Sun et al. 2023; Zhang et al. 2022a), salt (Kim et al., 2022; Yazdani et al. 2021), drought (Ali et al., 2022), and oxidative (Wang et al. 2015b) stresses (Fig. 3). The functions of OR family proteins under stress conditions can be associated with reactive oxygen species (ROS) scavenging and increased photosynthesis stability.

Reactive oxygen species such as singlet oxygen and hydrogen peroxide are normal byproducts of cellular respiration and other metabolic reactions. While typically at low levels, ROS accumulate during abiotic stress, potentially damaging macromolecules and causing cell death (Zhang et al. 2022a). Interestingly, plants overexpressing *Or* have higher expression and/or activity of ROS scavenging enzymes for example superoxide dismutase (SOD) and catalase (CAT) in drought-stressed Arabidopsis (Ali et al., 2022) and ascorbate peroxidase (APX2), CAT, and SOD-Cu/Zn in heat-stressed rice (Jung et al., 2021). This suggests that OR proteins may act as chaperones for these ROS scavenging enzymes, although a direct connection is yet to be established. As nonpolar compounds, carotenoids are excellent natural ROS scavengers (Shen et al. 2018). Crops overexpressing *Or* display an increased antioxidative activity in potatoes (Kang et al. 2023), lower levels of cell damage in rice (Jung et al., 2021), and enhanced radical scavenging activity in sweet potato callus (Kim et al., 2021). Thus, OR chaperones may safeguard ROS scavenging enzymes for radical detoxification in parallel with carotenogenic enzymes for increased antioxidant metabolites in a two-way stress protection. The physiological impact is increased tolerance to a variety of abiotic stress encounters, although the underlying mechanisms remain to be fully elucidated.

Carotenoid and chlorophyll pigments are essential to photosynthetic efficiency and maintenance. Plants under abiotic stress are well detailed to exhibit lower rates of photosynthesis due to impacts on related gene expression, protein stability, ROS accumulation, and CO_2_ capture, among others, potentially resulting in low yield and cell or plant death (Gururani et al. 2015; Muhammad et al. 2020). In sweet potato plants under heat stress conditions OR extends its chaperone capacity to PsbP, a subunit of photosystem II (PSII), to help stabilize PSII and maintain its photosynthetic capabilities, thereby reducing the detrimental impacts of abiotic stress (Kang et al. 2017a). Further, OR family proteins co-chaperone the biosynthesis of both chlorophyll and carotenoid photosynthetic pigments and safeguard a plant’s photosynthetic capacity in response to stress tolerance (Sun et al. 2023). This connection was observed in Arabidopsis and tomato under heat stress (Sun et al. 2023), Arabidopsis under oxidative, heat, drought, and salt stress (Kang et al. 2023), and in rice callus under heat stress (Jung et al., 2021).

Variable OR effects in different plant species possibly underly some of the conflicting information reported in the literature. For example, one group found that overexpression of *Or* in rice produced a variegated-like leaf phenotype alongside decreased tolerance to salt and cold stressors (Yu et al. 2021). The authors noted that this is likely due to most studies on OR family genes in dicots rather than monocots. However, their findings conflicted with other literature studying monocots (Jung et al., 2021; Kim et al., 2022). More interestingly is the dichotomy between the plethora of research studies positively linking OR^OR^ with plant growth and stress tolerance and those which indicate a negative connection. A recent Arabidopsis study shows that silencing of *Or* increases while its overexpression decreases leaf size (Wang et al. 2024). This is contradictory to previous literature which shows no differences in leaf size with overexpression and single knockout of *Or* family genes under both long- and short-day light conditions and significantly smaller leaves in the double knockout (Sun et al. 2023; Yuan et al. 2015a; Zhou et al. 2015), bringing to question the validity of genetic materials.

### Emerging roles of OR chaperones

The OR chaperone family in recent studies features their multifunctional roles in carotenoid accumulation, plastid biogenesis, stress tolerance, and as tools for crop biofortification. Despite over two decades of investigation, much remains to be discovered regarding their functions and protein targets.

OR chaperones belong to DnaJE1 proteins that are involved in numerous plastid-associated processes. Many members of DnaJE1 proteins are co-expressed with chloroplast-targeted proteins (Pulido & Leister 2018) and have roles in photosynthesis (Fristedt et al. 2014; Hartings et al. 2017; Lu et al. 2011; Zagari et al. 2017) and chloroplast development (Brutnell et al. 1999; Munoz-Nortes et al. 2017; Pfalz et al. 2006; Sun et al. 2019; Zagari et al. 2017), along with OR chaperones in carotenoid and chlorophyll biosynthesis (Sun et al. 2023; Zhou et al. 2015). Collectively, these diverse functions hint at the co-evolution of DnaJE1 proteins in accordance with development of the photosynthetic machinery and plastid metabolism pathways. This suggests that OR family proteins have the potential to directly interact with and modulate other plastid localized processes, pathways, and possibly beyond.

ORANGE family proteins interact with known protein targets (Fig. 3) through both chaperone and non-chaperone activity. PSY (Park et al. 2016; Rao et al. 2024; Zhou et al. 2015), FBN1 (Zhou et al. 2023), CHLI (Sun et al. 2023), and PsbP (Kang et al. 2017a) protein levels are postively regulated through OR interactions to influence carotenoid biosynthesis and storage, chlorophyll biosynthesis, and photosystem assembly. Non-chaperone associations with TCP14 (Sun et al. 2019), eRF1-2 (Zhou et al. 2011), TIC40 and TIC110 proteins (Yuan et al. 2021a), ARC3 (Sun et al. 2020b), and LHCB (Chayut et al. 2021) further connect the OR family as regulators in transcriptional regulation, protein import, and plastid division. Just within its current known set of protein targets, OR chaperones regulate diverse processes with significant influence on plant physiology, such as photosynthesis, plant growth, and stress tolerance. Further, *Or* has been reported for additional roles in affecting plant development including flowering time and fruit set (Yazdani et al. 2019). It is highly likely that more targets of OR family proteins will be discovered in the future. Depending on their targets, OR chaperones may link to different processes in plant growth and development to affect various agronomic traits beyond what are described here.

### Future perspectives and conclusions

DnaJE1 chaperone proteins present a unique opportunity for fine-tuning protein landscapes towards engineering plants with desirable physiological traits. Among them, the evolutionarily conserved OR family proteins have been connected to a plethora of core plant metabolism pathways and processes since its identification over two decades ago. These studies demonstrate this family’s preservation of functions for modulating photosynthetic capacity, nutrition, and stress tolerance in plants including algae, rice, carrot, melon, cauliflower, potato, and many more. Continued study of this group will no doubt connect them to additional plant pathways and processes critical for crop production and resilience. OR chaperones have already been used to engineer a variety of crop species with increased nutritional value and stress tolerance. Exciting roles of OR chaperones are expected to be discovered.

**Fig. 4:**
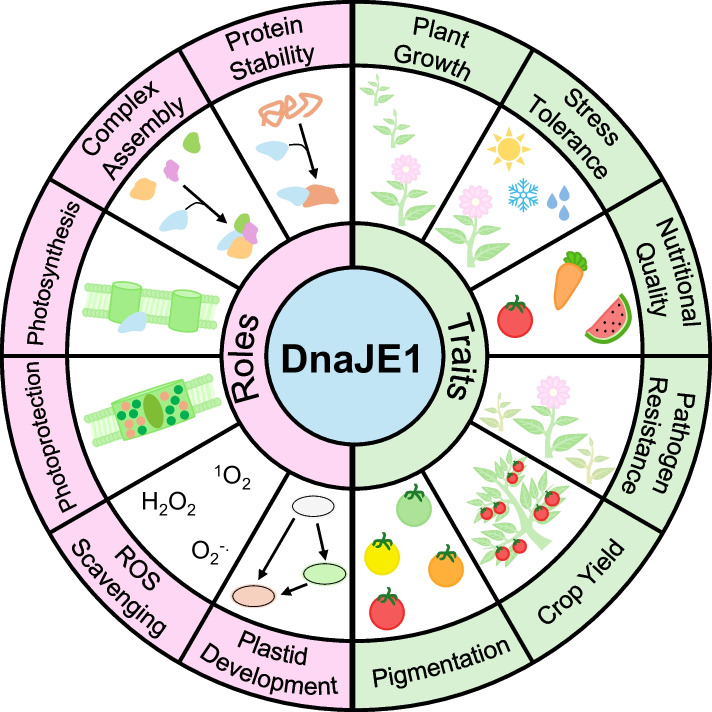
Known and speculative roles and traits of DnaJE1 chaperone proteins. DnaJE1 chaperones majorly localize to plastids and have roles in various processes (left, pink). These roles and functions influence numerous agronomically important traits in crops (right, green). Taken together, DnaJE1 chaperones modulate protein quality control and may provide pioneering genetic tools for niche physiological scale adjustments

In a wider scope, the study of the OR family as a group of DnaJE1 proteins expands our current understanding of molecular chaperones in the processes of plant metabolism, development, and resilience. Members of this conserved DnaJE1 group apply their basic functions of protein stability and complex assembly across numerous pathways with additional roles emerging (Fig. 4). They are documented to function in photosynthesis (Fristedt et al. 2014; Hartings et al. 2017; Lu et al. 2011; Zagari et al. 2017), chloroplast gene expression (Pfalz et al. 2006) and development (Brutnell et al. 1999; Munoz-Nortes et al. 2017; Sun et al. 2019; Zagari et al. 2017); carotenoid and chlorophyll biosynthesis (Lu et al. 2006; Sun et al. 2023; Zhou et al. 2015), pathogen resistance (Ham et al. 2006), and seedling establishment (Hartings et al. 2017), among others described above. At its core, DnaJE1 chaperones extend our knowledge of the plastid PQC where they directly influence various plant traits (Fig. 4). On the surface, chaperone proteins can and are being leveraged by plant breeders for building the next generation of agricultural crops with improved nutritional quality, photosynthetic efficiency, and environmental resilience.

## Data Availability

Not applicable.

## Abbreviations

ABA: Abscisic acid
DMAPP: Dimethylallyl diphosphate
GA: Gibberellin
GGPP: Geranylgeranyl phosphate
GRL: Glutaredoxin-like
IPP: Isopentyl diphosphate
PQC: Plastid quality control
PSII: Photosystem II
ROS: Reactive oxygen species
SL: Strigolactones
SNP: Single nucleotide polymorphism
VAD: Vitamin A deficiency
